# Association of periodontitis with oral malodor in Korean adults

**DOI:** 10.1371/journal.pone.0247947

**Published:** 2021-03-04

**Authors:** YoungHa Song, Yoo-Been Ahn, Myung-Seop Shin, David Brennan, Hyun-Duck Kim

**Affiliations:** 1 Australian Research Centre for Population Oral Health, Adelaide Dental School, The University of Adelaide, Adelaide, South Australia, Australia; 2 Department of Preventive and Social Dentistry, School of Dentistry, Seoul National University, Seoul, Korea; 3 Dental Research Institute, School of Dentistry, Seoul National University, Seoul, Korea; University Lyon 1 Faculty of Dental Medicine, FRANCE

## Abstract

This study aimed to evaluate the association of periodontitis with the organoleptic score (OLS)-defined oral malodor after validating OLS with odoriferous sulfur compounds in mouth air among Korean adults. A total of 330 adults aged 47–86 years were recruited from the Yangpyeong health cohort, South Korea, in 2015. Oral malodor was assessed using a 6-point OLS by a trained dentist and validated with the concentrations of hydrogen sulfide (HS) and methyl mercaptan (MM) using a gas chromatographer. Periodontitis was measured by assessing the radiographic alveolar bone loss on digital orthopantomography. Statistical analyses including descriptive statistics, partial correlation, ANOVA, and multivariable logistic regression with putative confounders were applied. OLS was significantly correlated with the concentrations of HS and MM (partial *r* = 0.401 and 0.392, respectively; both *p*<0.001) after controlling for confounders. Individuals with periodontitis had 1.8 times the risk of OLS-defined oral malodor in multivariable models (adjusted odds ratio = 1.77 in the model with the number of teeth and 1.82 in the model with denture wearing; *p* = 0.047 and 0.035, respectively). Periodontitis was associated with OLS-defined oral malodor among Korean adults independent of known confounders. Periodontal conditions should be considered for clinical practice and research of oral malodor.

## Introduction

Periodontitis is a multifactorial inflammatory disease of the tissues surrounding and supporting tooth structure, possibly leading to chronic and/or acute progressive destruction of the periodontium [1]. It is a highly prevalent oral health disease affecting approximately one-third of the adult population in the world [2] and Korea [3]. Periodontitis can cause subsequent oral health conditions including loose/drifting teeth and toothache, which consequently impacts oral health-related quality of life [4]. One of the symptoms closely relevant to psychosocial factors of periodontal patients is oral malodor [5]. Oral malodor, or halitosis in dental nomenclature, is multifactorial in its origin and results from an interplay of oral, systemic and psychosomatic elements [6]. It ranks as the third most frequent reason of patient’s visit to the dentist, behind dental caries and periodontal disease [7]. Oral malodor is of a significant health concern as it can impair an individual’s social interactions, quality of life, and potentially lead to low self-esteem or mood disorders such as depression [8].

Some of the Gram-negative periodontal pathogens in the subgingival plaque, saliva and tongue coating are known to produce volatile sulfur compounds (VSCs), such as hydrogen sulfide (HS) and methyl mercaptan (MM) [9]. They are the result of the microbial putrefaction of food debris, cells, saliva and blood [9]. An early in-vitro study demonstrated that specific periodontopathic bacteria namely, *T*. *denticola*, *P*. *gingivalis*, and *T*. *forsythia* are capable of producing large amounts of HS and MM, and are strongly related to increased periodontal pocket depth and bleeding on probing [10]. More recently, it was demonstrated that oral malodor is associated with the presence of periodontal bacteria in saliva [11] and *P*. *gingivalis* in tongue coating [12]. In addition, VSC levels in mouth air increase according to the severity of periodontitis [13, 14]. Similarly, VSC levels were elevated in deep periodontal pockets compared with shallow counterparts [15, 16].

Considering that periodontitis and oral malodor share certain microbiologic pathogenesis, the early detection of oral malodor could contribute to the evaluation of periodontal disease. However, few studies have employed both organoleptic scoring (OLS) and supplementary measurement of VSC concentrations for the potential association between periodontitis and oral malodor. In addition, few data have been available on the association of periodontitis with oral malodor after adjusting for relevant covariates. Driven by this research gap, this study aimed to explore the association of periodontitis with oral malodor in Korean adults. For rigorous results, we included putative confounders in the analysis and adopted the validation process of the OLS for oral malodor with the biochemical measurement of VSCs in mouth air.

## Materials and methods

### Ethical consideration

This study was approved by the Institutional Review Board of Seoul National University, School of Dentistry (IRB No: S-D20130005). All participants provided written informed consent after receiving a full explanation of the study details.

### Study design and participants

In this cross-sectional study, we recruited 330 participants for an oral assessment under a larger scale ‘Korean Genome and Epidemiology Study-Cardiovascular disease association study’ (KoGES_CAVAS) [17], conducted in Yangpyeong, Korea, in 2015. The KoGES_CAVAS is a community-based cohort study providing health examinations with follow-up programs for individuals aged 40 years or more, directed by the Korea Centers for Disease Control and Prevention. The cohort profile is available with information about sampling methods and survey contents elsewhere [17]. Detailed numbers and procedures of participant eligibility, recruitment, and assessment for this study are provided in the supplementary material with a flow diagram (S1 Fig). A standardized protocol was employed to train all interviewers and examiners for questionnaires and health assessment procedures. Out of the 330 participants enrolled, only those who had six or more natural teeth and complete datasets were included in the analysis, which constituted a total of 302 individuals, aged 47 to 86 years (Table 1). All of the valid participants were included in the *association* sample for exploring the association of periodontitis with organoleptic oral malodor. A subgroup of 111 participants were randomly selected as the *validation* sample for validating OLS of oral malodor with the concentration of odoriferous VSCs.

**Table 1:** Characteristics of the subjects by oral malodor (OLS≥2) in the association and validation samples.

Variable	Association sample (*n* = 302)			Validation sample (*n* = 111)		
	Oral Malodor		*p* value*	Oral Malodor		*p* value*
	No (*n* = 161)	Yes (*n* = 141)		No (*n* = 57)	Yes (*n* = 54)	
Periodontitis^†^, *n* (%)			**0.001**			0.100
No	**72 (44.7)**	**36 (25.5)**		22 (38.6)	13 (24.1)	
Yes	**89 (55.3)**	**105 (74.5)**		35 (61.4)	41 (75.9)	
Age, year (SD)	**63.4 (0.65)**	**66.3 (0.71)**	**0.003**	**62.5 (1.07)**	**68.8 (1.07)**	**<0.001**
Sex, *n* (%)			**0.015**			0.111
Female	**112 (69.6)**	**79 (56.0)**		40 (70.2)	30 (55.6)	
Male	**49 (30.4)**	**62 (44.0)**		17 (29.8)	24 (44.4)	
Education level, *n* (%)			**0.001**			**0.006**
≤ Middle-school	**84 (52.2)**	**101 (71.6)**		**29 (50.9)**	**41 (75.9)**	
≥ High-school	**77 (47.8)**	**40 (28.4)**		**28 (49.1)**	**13 (24.1)**	
Tooth brushing, *n* (%)			0.560			0.249
≤ 1 time per day	22 (13.7)	15 (10.6)		9 (15.8)	8 (14.8)	
2 times per day	80 (49.7)	78 (55.3)		21 (36.8)	28 (51.9)	
≥ 3 times per day	59 (36.6)	48 (34.0)		27 (47.4)	18 (33.3)	
Number of teeth (SD)	**23.2 (0.44)**	**21.5 (0.49)**	**0.011**	22.4 (0.81)	21.3 (0.80)	0.327
Denture-wearing, *n* (%)			0.249			0.703
No	133 (82.6)	109 (77.3)		45 (78.9)	41 (75.9)	
Yes	28 (17.4)	32 (22.7)		12 (21.2)	13 (24.1)	
Hyposalivation^‡^, *n* (%)			0.745			0.157
No	61 (37.9)	56 (39.7)		15 (26.3)	21 (38.9)	
Yes	100 (62.1)	85 (60.3)		42 (73.7)	33 (61.1)	
Tongue coating, *n* (%)			**<0.001**			**0.011**
No	**134 (83.2)**	**76 (53.9)**		**48 (84.2)**	**34 (63.0)**	
Yes	**27 (16.8)**	**65 (46.1)**		**9 (15.8)**	**20 (37.0)**	
Preference for onion, *n* (%)			0.628			0.307
No	80 (49.7)	74 (52.5)		23 (40.4)	27 (50.0)	
Yes	81 (50.3)	67 (47.5)		34 (59.6)	27 (50.0)	
Drinking, *n* (%)			0.289			0.650
Never	76 (47.2)	58 (41.1)		25 (43.9)	26 (48.1)	
Ever in lifetime	85 (52.8)	83 (58.9)		32 (56.1)	28 (51.9)	
Smoking, *n* (%)			0.085			0.352
Never	149 (92.5)	122 (86.5)		53 (93.0)	47 (87.0)	
Ever in lifetime	12 (7.5)	19 (13.5)		4 (7.0)	7 (13.0)	
Obesity^§^, *n* (%)			0.661			0.072
No	85 (52.8)	78 (55.3)		23 (40.4)	31 (57.4)	
Yes	76 (47.2)	63 (44.7)		34 (59.6)	23 (42.6)	
Diabetes^∥^, *n* (%)			**0.015**			0.058
No	**150 (93.2)**	**119 (85.1)**		51 (89.5)	41 (75.9)	
Yes	**11 (6.8)**	**22 (14.9)**		6 (10.5)	13 (24.1)	
Hypertension^¶^, *n* (%)			0.425			**0.046**
No	93 (57.8)	75 (53.2)		**34 (59.6)**	**22 (40.7)**	
Yes	68 (42.2)	66 (46.8)		**23 (40.4)**	**32 (59.3)**	
ENT diseases, *n* (%)			0.117			**0.034**
No	127 (78.9)	121 (85.8)		**44 (77.2)**	**50 (92.6)**	
Yes	34 (21.1)	20 (14.2)		**13 (22.8)**	**4 (7.4)**	
GI diseases, *n* (%)			0.152			0.113
No	119 (73.9)	114 (80.9)		39 (68.4)	44 (81.5)	
Yes	42 (26.1)	27 (19.1)		18 (31.6)	10 (18.5)	

SD: standard deviation; ENT: ear-nose-throat; GI: gastrointestinal.

*Obtained from chi-square or Student T-test for all variables except for smoking and ENT diseases with Fisher’s exact test

^†^Periodontitis: two or more interproximal sites with radiographic alveolar bone loss ≥4mm.

^‡^Hyposalivation: unstimulated salivary flow rate ≤ 0.1mL/min.

^§^Obesity: body mass index (kg/m^2^) ≥ 25.

^∥^Diabetes: fasting plasma glucose ≥126 mg/dL or diagnosed or being medicated for diabetes.

^¶^Hypertension: SBP≥140mmHg or DBP≥90mmHg or diagnosed or medicated for hypertension.

Bold denotes statistical significance at *p* <0.05.

### Assessment of oral malodor

The overall protocol of organoleptic assessment followed the recommendations for dental professionals by Greenman *et al*. [18]. Participants were asked to refrain from any oral activities through mouth which may cause instant/tentative changes on oral malodor including but not limited to eating, drinking, smoking, and using mouthwash for three hours before the assessment of oral malodor. After 3 minutes of keeping the mouth closed by avoiding speaking, participants were instructed to release mouth air slowly through a 10cm-long paper tube set in the center of a privacy screen (30×20cm). The evaluation of OLS was conducted by a single examiner (Dr. MS Shin), a dentist pre-trained for this procedure as was demonstrated with sufficient discrimination in previous trials of double-blinded and randomized design [18, 19]. The examiner assessed the exhaled breath at a distance of approximately 10cm away from the paper tube (diameter 3cm), hence total 20cm away from the examinee. Oral malodor was assessed using a 6-point scale (0: no odor detectable; 1: barely noticeable odor; 2: slight odor; 3: moderate odor; 4: strong odor; 5: extremely strong odor) [20, 21] and classified into dichotomous groups: oral malodor negative (OLS 0–1) and positive (OLS 2–5). In order to standardize the OLS assessment, the organoleptic judge was trained for organoleptic scoring by assigning specific threshold concentrations of HS and MM for each score, prior to the assessment. The corresponding threshold concentrations for each increasing score of OLS were decided by three researchers (Drs. HD Kim, MS Shin and Y Choi-the principal developer of TwinBreasor^®^). The concentrations for each OLS score were from multiple organoleptic testing of pure gas: <65, ≥65, ≥130, ≥260, ≥520, ≥2100 parts per billion (ppb) for HS, and <12, ≥12, ≥25, ≥50, ≥100, ≥200 ppb for MM, respectively from 0 to 5. For participants in the validation sample, HS and MM levels in mouth air were measured by a portable gas chromatographer (TwinBreasor^®^, IsenLab, Gyeonggido, Korea), equipped with a flame photometric detector. As 10mL sample of participant’s mouth air passed through an electrolytic sensor, the concentrations of HS and MM were detected indicating a peak level in ppb on the digital scale of the monitor.

### Assessment of periodontitis

Periodontal status of the participants was assessed by a single dentist (Dr. YB Ahn) using orthopantomography taken by a digital panoramic tomography machine (Pax-Primo^®^, Vatech Global, Seoul, Korea). We used the radiographic alveolar bone loss (RABL) as a surrogate of clinical attachment loss (CAL) to indicate periodontal status, for practical applicability of radiographic methods [22, 23] and situational infeasibility of measuring CAL in this field study. The RABL, defined as the vertical distance between the cemento-enamel junction (CEJ) and the deepest point of the alveolar bone crest, was measured on the mesial and distal side of all teeth. When CEJ was not clearly visible for technical reasons (overlapping teeth or prosthesis), CEJ was determined by anatomical supposition by referring to the adjacent teeth. The radiographic assessor was blinded to the oral malodor status of the examinee. Classification of periodontitis status followed the CAL guideline of the CDC/AAP case definitions for surveillance of periodontitis [24]. Since the number of cases with severe periodontitis was small following the guideline (*n* = 35), periodontal status was dichotomized into periodontitis no (no or mild periodontitis) and yes (moderate and severe periodontitis; RABL ≥4mm at two or more interproximal sites, not on the same tooth).

### Assessment of potential confounders

The authors considered the following as potential confounders for oral malodor: age, sex, education level, tooth-brushing frequency, number of teeth, denture-wearing, hyposalivation, tongue coating, dietary preference for onion, drinking alcohol, smoking, obesity, diabetes, hypertension, ear-nose-throat (ENT) and gastrointestinal diseases [25, 26].

Information on socio-demographics, daily tooth-brushing frequency, food preference, smoking, alcohol intake, medical history and medications was collected using a questionnaire administered by trained interviewers. Participants’ age was coded as a continuous variable by year and education level was dichotomized as being middle-school or lower and high-school or higher. The tooth-brushing frequency was coded into tertiles: once or less, twice, and three times or more daily. The number of natural teeth except for third molars and root fragments was counted in the orthopantomography by the radiographic assessor, as is described in the assessment of periodontal status. Denture-wearing status and tongue coating was assessed by a trained dentist. Tongue coating was assessed from the dorsal tongue surface divided by a 3×3 grid for scoring three levels of coating thickness. The criterion of one or more out of 9 sections with a thick-coating score dichotomized tongue coating into the presence or absence of the variable [27]. Salivary flow rate was measured by collecting unstimulated saliva in milliliter for 10 minutes. Hyposalivation was defined as having a salivary flow rate ≤0.1mL/min [28]. Dietary preference for onion was obtained by the question: ‘Have you had onion-containing food within the last 24 hours?’ Self-reported smoking status was dichotomized into never or ever being a smoker in lifetime. Self-reported alcohol consumption was similarly coded into none or ever in lifetime. The dichotomy with the past experience was due to the low prevalence of current smoking and drinking in our study sample. For obesity, body weight and height were measured to the nearest 0.1kg and 0.1cm, respectively, with the participants in light indoor clothing without shoes. Body mass index (BMI) was calculated using the formula weight/height^2^ (kg/m^2^). Obesity was defined as having BMI over 25 kg/m^2^ [29]. To measure diabetic conditions, blood samples were collected from the antecubital vein of each participant during the morning after 8-hour overnight fasting. Fasting plasma glucose (FPG) levels were analyzed from the blood sample on the same day using an automatic analyzer (ADVIA1650 Chemistry Analyzer^®^, Siemens, New York, NY). Diabetes was defined as FPG over 126mg/dL or diagnosed/medicated for diabetes, according to the WHO criteria [30]. A standard mercury sphygmomanometer (Baumanometer^®^, W.A. Baum, Copiague, NY) was used to measure the blood pressure (BP) on the participants’ right arm in a seated position after resting at least five minutes before the initial measurement. Systolic and diastolic BP were measured twice to the nearest 2mmHg at a five-minute interval and the average values were used. For the purpose of our analyses, hypertension was defined as having an average systolic BP over 140mmHg or diastolic BP over 90mmHg [31], or being medicated for hypertension. Information about ENT and gastrointestinal diseases was collected by self-reported experience from the questionnaire.

### Statistical analyses

In the analysis, the outcome variable was oral malodor and the main explanatory variable was periodontitis status. Descriptive statistics of the participants’ characteristics were analyzed as frequencies and proportions. Statistically significant differences between the groups of positive and negative oral malodor in terms of variables included in the analysis were examined using the chi-square test and independent T-test.

The correlation between OLS and concentrations of VSCs in mouth air was evaluated by calculating spearman’s correlation coefficients for each component, HS and MM. This correlation was re-examined after controlling for the aforementioned confounders to calculate partial *r* coefficients for HS and MM against OLS. In order to assess the validity of OLS method, estimated marginal mean concentrations of HS and MM for each OLS group were calculated by adjusting for confounders among the validation sample. In the process, OLS 3, 4 and 5 groups were combined into one 3+ group (*n* = 28) as the number of participants in the OLS 4 (*n* = 6) and 5 (*n* = 2) category was small for the sensitivity of dose-response relationship. Differences among the four OLS groups for statistical significance were assessed by analysis of covariance (ANCOVA) and multiple comparison analysis of Bonferroni. The validity of OLS was further examined by assessing the adjusted relationship between OLS-defined oral malodor (OLS≥2) and oral malodor parameters including the OLS and concentrations of HS and MM. Multivariable logistic regression analysis was applied to evaluate the adjusted odds ratios (aOR) for the association of periodontitis with oral malodor. The logistic regression was performed in two models including either the number of teeth for its significant association with oral malodor [32, 33] or denture wearing for its high collinearity with the number of teeth (Spearman’s *ρ* = -0.616, *p*<0.001 in this study sample). Finally, ANCOVA was conducted to assess the adjusted relationship between periodontitis and OLS, the concentration levels of HS and MM and MM/HS ratio.

## Results

### General characteristics of the participants

Among the 302 participants with six or more natural teeth, the prevalence of oral malodor was 46.7% (Table 1). In the association sample, a significantly higher prevalence of oral malodor was observed in those with periodontitis, in older age, male, lower education, low number of teeth, tongue coating, and diabetes. Similarly, in the validation sample, a significantly higher prevalence of oral malodor was observed in participants of older age, in lower education, with tongue coating, hypertension, and ENT diseases. The mean OLS for association and validation samples were not significantly different (1.54 versus 1.65; *p* = 0.407). Some characteristics of the validation sample differed from that of the association sample, including a higher prevalence of hypertension and a lower prevalence of ENT diseases among oral malodor positives.

### Validation of the organoleptic oral malodor score against VSC concentrations

**Fig 1:**
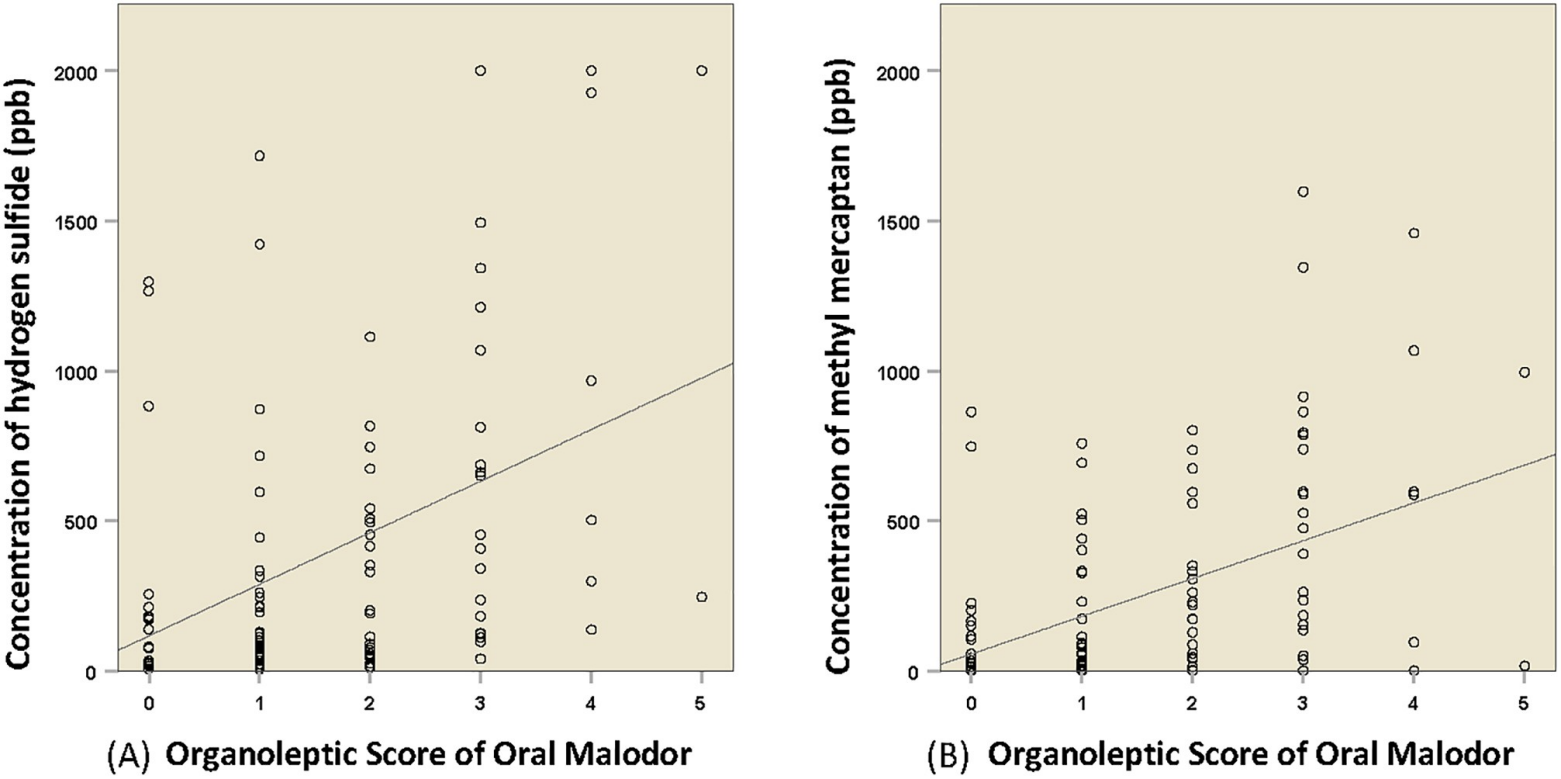
Correlation between organoleptic score (OLS) for oral malodor and the concentrations of volatile sulfur compounds (VSC) (*n* = 111). (A) correlation between OLS and hydrogen sulfide level (ppb) in mouth air (Spearman’s *ρ* = 0.386, *P*<0.001; partial r* = 0.401, *P*<0.001); (B) correlation between OLS and methyl mercaptan level (ppb) in mouth air (Spearman’s *ρ* = 0.385, *P*<0.001; partial r* = 0.392, *P*<0.001). *Adjusted for age, sex, education level, tooth-brushing frequency, denture-wearing, periodontitis, hyposalivation, tongue coating, preference for onion, drinking, smoking, obesity, diabetes, hypertension, ear-nose-throat diseases and gastrointestinal diseases. Solid line represents the linear relationship of the correlation.

**Fig 2:**
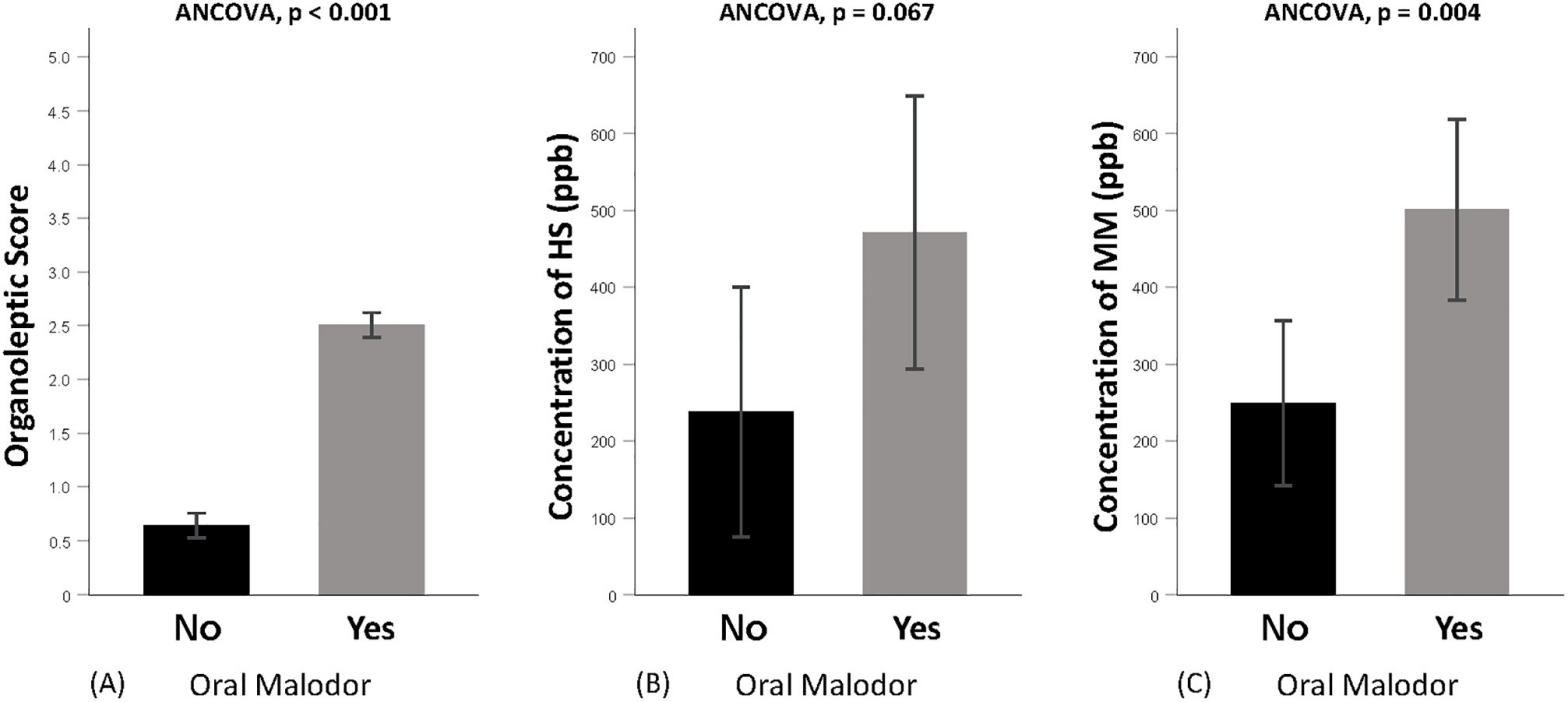
Adjusted relationship between oral malodor (OLS≥2) and oral malodor parameters (*n* = 111). (A) organoleptic score; (B) concentration of hydrogen sulfide (HS); (C) concentration of methyl mercaptan (MM). Each bar and error lines represent the mean ± standard error for oral malodor no and yes groups. *P* values are obtained from ANCOVA adjusted for age, sex, education level, tooth-brushing frequency, denture-wearing, periodontitis, hyposalivation, tongue coating, preference for onion, drinking, smoking, obesity, diabetes, hypertension, ear-nose-throat diseases and gastrointestinal diseases.

The concentrations of HS and MM were significantly correlated with OLS with both Spearman’s and partial correlation coefficients (Fig 1). This correlation was consistent for HS and MM after adjusting for confounders (Table 2). For those assessed with higher OLS ≥3, the mean concentration of HS and MM in mouth air was significantly higher than those with lower OLS. In addition, the adjusted mean OLS was significantly higher for the oral malodor positive group than the negative group controls (mean ± standard error [SE]: 2.51±0.12 versus 0.65±0.11, ANCOVA, *p*<0.001) (Fig 2). Similarly, the significant association of higher VSC concentration with oral malodor was consistent for MM (mean ± SE: 501.44±117.65 versus 249.56±107.49, ANCOVA, *p* = 0.004) and marginally lost for HS (mean ± SE: 471.86±177.70 versus 237.73±162.34, ANCOVA, *p* = 0.067) after adjusting for confounders.

**Table 2:** Validation of the Organoleptic Scoring (OLS) of oral malodor against hydrogen sulfide (HS) and methyl mercaptan (MM) concentrations in mouth air (*n* = 111).

OLS	Description	*N*	HS, ppb (mean±SE)	*P* value*	MM, ppb (mean±SE)	*P* value*
0	No odor detectable	20	341.55 ± 198.55^ab^	**0.015**	352.43 ± 133.57^ab^	**0.004**
1	Barely noticeable odor	37	285.94 ± 165.98^a^		255.01 ± 111.66^a^	
2	Slight odor	26	259.63 ± 186.77^ab^		383.72 ± 125.65^ab^	
3+	≥ Moderate odor	28	746.77 ± 198.17^b^		647.38 ± 133.32^b^	

SE: standard error of the mean.

*P values are obtained from ANCOVA adjusting for age, sex, education level, tooth-brushing frequency, denture-wearing, periodontitis, hyposalivation, tongue coating, preference for onion, drinking, smoking, obesity, diabetes, hypertension, ear-nose-throat diseases and gastrointestinal diseases.

Superscript denotes significantly different groups at *p* <0.05 by multiple comparison analysis of Bonferroni.

Bold denotes statistical significance at *p* <0.05.

### Association of periodontitis with oral malodor

**Fig 3:**
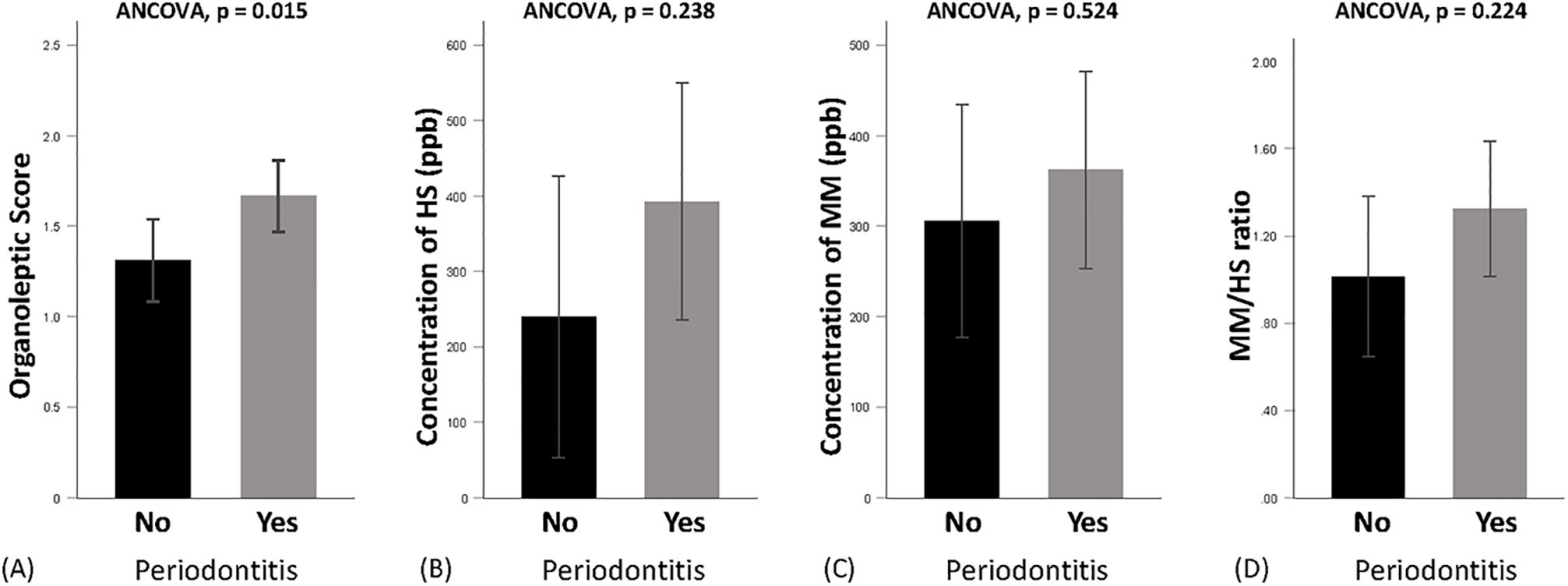
Adjusted relationship between periodontitis and oral malodor parameters. (A) organoleptic score (*n* = 302); (B) hydrogen sulfide (HS) level (*n* = 111); (C) methyl mercaptan (MM) level (*n* = 111); (D) MM/HS ratio (*n* = 111). Each bar and error lines represent the mean ± standard error for periodontitis no and yes groups. *P* values are obtained from ANCOVA adjusted for age, sex, education level, tooth-brushing frequency, denture-wearing, hyposalivation, tongue coating, preference for onion, drinking, smoking, obesity, diabetes, hypertension, ear-nose-throat diseases and gastrointestinal diseases.

Those with periodontitis had 1.8 times the risk of oral malodor compared to those without (aOR = 1.77 in the model with the number of teeth and 1.82 with denture wearing variable; *p* = 0.047 and 0.035, respectively) (Table 3). In addition, the adjusted mean OLS was significantly higher in participants with periodontitis compared to periodontally healthy controls (mean ± SE: 1.67±0.20 versus 1.31±0.23, ANCOVA, *p* = 0.015) (Fig 3). Similarly, the concentrations of HS, MM and the MM/HS ratio were higher in the periodontitis group, yet such differences were not statistically significant (mean ± SE: 392.50±156.98 versus 239.66±186.38, ANCOVA, *p* = 0.238 for HS; 362.22±108.50 versus 305.47±128.82, ANCOVA, *p* = 0.524 for MM; 1.32±0.31 versus 1.01±0.37, ANCOVA, *p* = 0.224 for MM/HS ratio).

**Table 3:** Adjusted association of periodontitis with oral malodor (OLS ≥2) in association sample (*n* = 302).

Variables	*N*	*Model 1*. *with number of teeth*		*Model 2*. *with denture wearing*	
		Odds ratio (95% CI)*	*p* Value	Odds ratio (95% CI)*	*p* Value
Periodontitis^†^					
No	108	1		1	
Yes	194	**1.77 (1.01–3.10)**	**0.047**	**1.82 (1.04–3.18)**	**0.035**
Age	302	0.99 (0.96–1.04)	0.778	1.00 (0.96–1.04)	0.927
Sex					
Female	191	1		1	
Male	111	1.66 (0.85–3.25)	0.137	1.64 (0.83–3.21)	0.153
Education level					
≤ Middle-school	185	1		1	
≥ High-school	117	**0.40 (0.21–0.75)**	**0.004**	**0.37 (0.20–0.70)**	**0.002**
Tooth brushing frequency					
≤ 1 time per day	37	1		1	
2 times per day	158	1.86 (0.82–4.24)	0.139	1.89 (0.82–4.34)	0.135
≥ 3 times per day	107	1.86 (0.78–4.44)	0.164	1.95 (0.81–4.72)	0.139
Number of teeth	302	1.00 (0.95–1.05)	0.884	–	–
Denture-wearing					
No	242	–	–	1	
Yes	60	–	–	0.66 (0.33–1.32)	0.237
Hyposalivation^‡^					
No	123	1		1	
Yes	179	0.96 (0.57–1.63)	0.879	0.94 (0.55–1.58)	0.802
Tongue coating					
No	210	1		1	
Yes	92	**3.52 (1.90–6.51)**	**<0.001**	**3.64 (1.96–6.75)**	**<0.001**
Preference for onion					
No	154	1		1	
Yes	148	0.85 (0.51–1.44)	0.553	0.82 (0.49–1.38)	0.453
Drinking					
Never	134	1		1	
Ever in lifetime	168	1.20 (0.69–2.09)	0.511	1.25 (0.71–2.18)	0.438
Smoking					
Never	271	1		1	
Ever in lifetime	31	0.85 (0.34–2.13)	0.720	0.84 (0.33–2.14)	0.720
Obesity^§^					
No	163	1		1	
Yes	139	0.89 (0.52–1.53)	0.672	0.88 (0.51–1.52)	0.653
Diabetes^∥^					
No	269	1		1	
Yes	33	**2.37 (1.01–5.57)**	**0.048**	2.28 (0.97–5.37)	0.058
Hypertension^¶^					
No	168	1		1	
Yes	134	0.95 (0.53–1.70)	0.862	0.93 (0.52–1.66)	0.809
ENT diseases					
No	248	1		1	
Yes	54	0.67 (0.34–1.33)	0.253	0.64 (0.32–1.27)	0.202
GI diseases					
No	233	1		1	
Yes	69	0.73 (0.39–1.40)	0.345	0.73 (0.38–1.39)	0.338

*Adjusted for age, sex, education level, tooth-brushing frequency, hyposalivation, tongue coating, preference for onion, drinking, smoking, obesity, diabetes, hypertension, ear-nose-throat diseases and gastrointestinal diseases.

^†^Periodontitis: two or more interproximal sites with radiographic alveolar bone loss ≥4mm.

^‡^Hyposalivation: unstimulated salivary flow rate ≤ 0.1mL/min.

^§^Obesity: body mass index (kg/m^2^) ≥ 25.

^∥^Diabetes: fasting plasma glucose ≥126 mg/dL or diagnosed or being medicated for diabetes.

^¶^Hypertension: SBP≥140mmHg or DBP≥90mmHg or diagnosed or medicated for hypertension.

Bold denotes statistical significance at *p* <0.05.

## Discussion

Our results demonstrated that periodontitis is associated with organoleptic oral malodor among Korean adults, independent of putative confounders. The validated organoleptic oral malodor was associated with those in periodontal conditions by being about 77~82% higher than those with no periodontitis after adjusting for covariates including age, sex, education level, tooth-brushing frequency, number of teeth or denture-wearing, hyposalivation, tongue coating, dietary preference for onion, drinking, smoking, obesity, diabetes, hypertension, ENT and gastrointestinal diseases.

Although mixed results of the associations between periodontitis and oral malodor have been reported [6], this study found a significant association supported by previous studies with common microbiologic pathogenesis. The progression of periodontitis can result in deepening periodontal pockets in which specific periodontopathogenic microorganisms can thrive and accelerate the production of VSCs such as HS and MM [1, 7]. Another plausible reason for the increased oral malodor in periodontal disease is the inflammation-associated hyperemia of the gingival tissue. A previous study demonstrated that the breakdown of blood components in the sulcular space may accelerate VSC production [34]. It is also reported that HS can inhibit the activities of cytochrome *c* oxidase and superoxide dismutase in gingival fibroblasts, leading to markedly increased levels of reactive oxygen species which in turn, activates caspase-9 and -3 and p53 causing apoptosis in periodontal tissue and osteoblasts [35].

Among diverse covariates included in the multivariable regression analysis, tongue coating, education level and diabetes were independently associated with oral malodor. Tongue coating is generally accepted as a primary source of VSC production in the oral cavity particularly with the size and contexture of tongue dorsum surface [6, 7]. Tongue coating is comprised of desquamated epithelial cells, blood cells and bacterial species [36]. Previous studies demonstrated that highly varied and diverse tongue microflora including specific periodontal pathogens such as *P*. *gingivalis*, *T*. *forsythia*, *P*. *intermedia* and *P*. *nigrescens* were associated with oral malodor and VSC production in periodontitis cases [11, 36, 37]. The association of education level with oral malodor can be attributed to the difference in oral health literacy and behaviors for health promotion [38]. The level of education has been reported as one of the social determinants of oral health including the prevalence of dental caries, periodontal conditions, and subjective oral health [39, 40] as well as oral malodor [38]. Diabetes has also appeared to be one of the possible systemic causes for oral malodor [26] as is reported in the study with a significant association in spite of less consistent results across models.

Some limitations of the study are worth noting. By the nature of cross-sectional design, findings from the study should be interpreted with caution as associations among variables, not necessarily as causal inferences. For example, oral hygiene behaviors including tooth brushing and smoking were not significantly or reversely associated with oral malodor. One of possible reasons for the association can be reverse causality that oral malodor may encourage the need of tooth brushing or be camouflaged by smoking, which warrants prospective longitudinal study design for the future. There also remains a risk of measurement bias for both explanatory and outcome variables. In this study, periodontal status, the explanatory variable, was classified by indirectly measuring RABL as a proxy for clinical periodontal probing. Despite applying a highly calibrated radiographic equipment, surveying periodontal disease based on RABL can be considered less advisable under the revised CDC/AAP classification framework of periodontitis [41]. We might have missed initial periodontitis cases and consideration for clinical periodontal pocket depth reflecting the activity of inflammation, which are not sensitive to radiographic methods leading to the dilution of estimated associations towards the null hypothesis. In addition, assessing variables by a single examiner may not guarantee the reliability of measurement despite standardized training and validating the assessment procedure before the field survey. Particularly, notwithstanding demonstrated discrimination by a single trained odor judge under well-controlled experimental conditions [18], OLS rated once by a single dentist in the field survey might have raised both intra- and inter-rater reliability issues for a rigorous study considering the subjective nature of oral malodor [7]. In future studies, for that matter, a more sophisticatedly designed method including consensus-based measurement by multiple examiners is advised for validity and reliability. For the characteristic of participants, sampling only from the age group over 40 years limited the exploration of the association in young adults who may have specific oral malodor-associated periodontal diseases such as acute necrotizing ulcerative gingivitis. Also, older adults are more likely to have been exposed to various oral malodor-associated risk factors for a longer period. Hence, it is possible that unforeseen confounding factors other than those included in our model could have contributed to the association.

Despite these limitations, our study has some strengths. First, the participants of this study were recruited from the community-dwelling general population as opposed to dental patients, which reduces the chance of selection bias and enhances the generalizability of the results. For instance, a previous cross-sectional study with a relatively large sample size was conducted among 823 individuals with periodontal diseases, but all of whom were patients at an oral malodor clinic [42]. Second, we present the results as adjusted odds ratios from multivariable regression models, which enables assessing the adjusted association of periodontitis with oral malodor independent of the confounding effects of relevant covariates. Finally, OLS was validated with VSC concentrations in pure gas and mouth air, which supported rigorous assessments and reduced classification bias of oral malodor.

This study provides practical implications for clinical practice and further research. The finding of the study highlights the practicability of organoleptic oral malodor assessment and possibility of periodontal health as a clinical indicator of oral malodor. By extension, oral malodor could be an indicator of the risk of developing relevant systemic diseases including cardiovascular diseases and diabetes considering their possibly communal composition of bacteria in etiology [43]. Despite the technological developments in gas chromatography, the organoleptic judgment remains the clinical “gold standard” in measuring oral malodor [7]. Considering subjective complaints [7] and multifactorial etiology [6] of oral malodor, measuring levels of VSC alone may not sufficiently or comprehensively assess the impact of oral malodor not least in the social context. In addition, relatively large variations of VSC concentrations from each individual’s circadian fluctuations [44] and technique-sensitivity of chromatographic measurement [45] renders the biochemical measurement less reliable for its application as an indicator of periodontal or systemic health. In fact, observed means of HS and MM concentrations for each OLS group among the validation sample (Table 2) were generally higher than pre-set threshold values described in the methods section. This could be due to the masking effect by the mixture of various odoriferous compounds in the halitotic breath, suggesting the concentration of odorants in the mouth air should exceed their experimental threshold [46]. Despite the training and standardization process of OLS assessment, the judge dentist’s fatigue/desensitization and following changes of objectionability could have increased the threshold levels for determining scores. Regarding VSC concentrations of each OLS score, we adopted geometric increases of threshold by 2–4 times based on the previous findings with statistically significant differences [47]. However, human olfaction intensity is known to increase logarithmically according to the Weber-Fechner law and Greenman *et al*. reported 4.2- and 7.2-fold increase in concentration to one increment of OLS for HS and MM [21]. Thresholds of odor have been reported in varied levels among studies in different measurement settings and ethnic/cultural groups [21, 46, 47], thus further studies for reliable thresholds in accordance with contextual difference are encouraged. In particular, the classification of oral malodor may need to be revisited for the presence from OLS 3 or higher based on VSC concentrations rather than the conceptual definition from OLS 2 or higher (Table 2). Organoleptic judgment, nonetheless, showed significant correlations with concentrations of individual odoriferous compounds of HS and MM. The results of our study imply that OLS can be a feasible parameter as suggested to be a clinical standard by previous studies [18, 45].

## Conclusions

Periodontitis was associated with oral malodor among Korean adults independent of known confounders. Dentists should take periodontal conditions into account for preventing or controlling dental patient’s oral malodor. Likewise, physicians are advised to provide care for oral malodor patients with the possibility of periodontitis as a source of the malodor. Further research with a prospective or experimental design will aid the clarification of the causality and mechanism of the association. Meanwhile, our data corroborate the accumulating evidence that oral malodor is not merely a social handicap but a potentially associated factor with periodontal disease status.

## Supporting information

S1 Fig Flow diagram of participants sampling in the study. (DOCX)

S1 Table Inclusion and exclusion criteria. (DOCX)

S1 Data (SAV)
