# Supplementary material for: Association of periodontitis with oral malodor in Korean adults
Source: PLoS One. 2021 Mar 4;16(3):e0247947. doi: 10.1371/journal.pone.0247947 (PMC7932065; doi:10.1371/journal.pone.0247947)
Supplement: S1 Fig — (DOCX) [file pone.0247947.s001.docx]

**S1 Fig.** Flow diagram of participants sampling in the study

Yangpyeong Dental Cohort Baseline, 2010-2014 (n= 1854)

## Actual sample

## Enrolment sample

## Baseline sample

Excluded through 1^st^ 5-year follow-up

(n= 50)

♦  Loss to follow up (n= 50)

Excluded by random selection

(n= 191)

Validation study participants

(n= 111)

Excluded by inclusion criteria (n= 28)

♦  Had less than six natural teeth

(n= 28)

Association study participants

(n= 302)

Yangpyeong Dental Cohort, 2015

(n= 330)

Yangpyeong Dental Cohort, 2010

(n= 380)
