# Supplementary material for: Association of periodontitis with oral malodor in Korean adults
Source: PLoS One. 2021 Mar 4;16(3):e0247947. doi: 10.1371/journal.pone.0247947 (PMC7932065; doi:10.1371/journal.pone.0247947)
Supplement: S1 Table — (DOCX) [file pone.0247947.s002.docx]

**S1 Table.** Inclusion and exclusion criteria

| Inclusion criteria | Exclusion criteria |
| --- | --- |
| Adults aged over 40 years living in Yangpyeong, Korea | Children and young adults aged younger than 40 years |
| Have six or more natural teeth | Have less than six natural teeth |
| Generally healthy with only history of chronic systemic disease (e.g. controlled DM or hypertension) | Systemic conditions with acute symptoms requiring possible hospitalisation |
| Intend to cooperate the protocol of assessing oral malodour by avoiding eating/drinking for three hours | Unable or not intend to cooperate the protocol of assessing oral health |
| Can understand and fill in questionnaires and informed consent | Refuse or incompletely submit questionnaires and informed consent |
